# Chemotherapy-induced peripheral neuropathy and social interaction anxiety in breast cancer patients: a cross-sectional study

**DOI:** 10.1186/s12905-026-04613-w

**Published:** 2026-06-16

**Authors:** Lu Xu, Jinghui Fan, Jianying Wang, Yan Tang, Na Li, Jiayang Liu, Wenjia Zhang

**Affiliations:** Renmin Hospital of Wuhan University, Wuhan, China

**Keywords:** Breast cancer, Chemotherapy-induced peripheral neuropathy, Disabilities of the Arm, Shoulder and Hand, Social Interaction Anxiety, Neutrophil-to-Lymphocyte Ratio, Moderated mediation model

## Abstract

**Background:**

Breast cancer patients face numerous physical and psychological challenges. Social engagement and psychological adjustment are critical to the quality of life throughout the survival phase. Although chemotherapy-induced peripheral neuropathy and physical handicap are linked to social adjustment, the interplay between these factors in breast cancer patients is unclear.

**Methods:**

This cross-sectional study was conducted between March and September 2025, recruiting 285 breast cancer patients from a Grade A tertiary hospital in central China. Recruitment was conducted using convenience sampling. Data were collected via a structured questionnaire comprising several sections: a demographic and clinical information questionnaire, Chemotherapy Induced Peripheral Neuropathy Assessment Tool、Quick Disabilities of the Arm, Shoulder and Hand Questionnaire、Social Interaction Anxiety Scale. Statistical analysis was performed using SPSS 27.0, including descriptive statistics, univariate analysis and correlation analysis. SPSS PROCESS macro models 4 and 7 were utilised to test the hypothesised pathways between variables.

**Results:**

The majority of patients were aged between 40 and 60; the mean ± standard deviation for social interaction anxiety scores was 19.91 ± 13.35.

Chemotherapy-induced peripheral neuropathy, as well as disabilities of the arm, shoulder and hand, were found to have a significant positive correlation with social interaction anxiety (all *P* < 0.05). The results of the mediation and moderation model indicated that disabilities of the arm, shoulder and hand mediated the relationship between chemotherapy-induced peripheral neuropathy and social interaction anxiety, with a significant indirect effect (95% CI: 0.003 to 0.025), accounting for 18.06% of the total effect. The association between chemotherapy-induced peripheral neuropathy and disabilities of the arm, shoulder and hand may be negatively modulated by the neutrophil-to-lymphocyte ratio (95% CI: -0.042 to -0.005).

**Conclusion:**

There is a direct link between chemotherapy-induced peripheral neuropathy and social interaction anxiety in breast cancer patients, with disabilities of the arm, shoulder and hand acting as a mediator. Focusing on the management of chemotherapy-induced peripheral neuropathy and disabilities of the arm, shoulder and hand may help this population achieve better psychosocial adaptation. Healthcare professionals should think about combining these elements into long-term health management for their patients. However, the negative moderating effect of neutrophil-to-lymphocyte ratio on the link between chemotherapy-induced peripheral neuropathy and disabilities of the arm, shoulder and hand is minor; more scientific research is needed to confirm its clinical significance.

## Introduction

### Background

According to the most recent data published by the International Agency for Research on Cancer (IARC) in 2022, there are 2.31 million new cases and 0.67 million fatalities of female breast cancer worldwide, with the incidence rate and mortality ranking second and fourth, respectively [1]. In recent years, the five-year survival rate for breast cancer patients in China has exceeded 60% [2]. The quality of life for patients with breast cancer is being impacted more and more by treatment-related adverse effects as survival times increase. Chemotherapy and surgical tumor resection are the mainstays of complete treatment; patients frequently develop treatment-related adverse physical reactions and complications, such as Chemotherapy-induced peripheral neuropathy (CIPN) and Disabilities of the Arm, Shoulder and Hand (DASH) [3–4]. In addition to physical symptoms, psychological concerns such as Social Interaction Anxiety (SIA) have emerged as key health management goals for breast cancer survivors [5]. Paying attention to the physical and mental health requirements of breast cancer patients has emerged as a public health priority in women’s oncology.

CIPN is one of the most common toxic side effects of chemotherapy [3]. According to reports, 70% of individuals with breast cancer may develop CIPN, and the symptoms may last for months or even years following chemotherapy. CIPN is defined by sensory, motor, and autonomic nerve dysfunction; it causes limb numbness, tingling, and sensory abnormalities, etc., due to injury to sensory and motor nerve fibers. This has a significant impact on patients’ quality of life and treatment adherence [6]. Severe CIPN also frequently leads to modifications in chemotherapy doses or even the forced stoppage of treatment, and is closely related to treatment results [7]. For chemotherapy breast cancer patients, constant monitoring and control of CIPN is critical.

Surgical tumor resection is still the cornerstone of breast cancer treatment, but surgery often damages the integrity of the affected pectoral muscle and armpit. According to research, up to 70% of breast cancer patients will experience symptoms such as lymphedema, limb numbness, pain, and limited shoulder movement within three years following surgery [8], which have a substantial impact on daily function and quality of life. This physical function impairment may extend beyond the physiological scope and into the socio-psycho-behavioural field, such as affecting the patient’s limb expression in social interaction, limiting daily social involvement, and other behaviors [9].

One of the most common psychological and behavioural concerns among breast cancer patients is SIA, which presents as negative emotional experiences—such as tension, avoidance, and worry—arising from a perceived degradation of one’s self-image or physical limits in social circumstances [10]. Patients may consciously avoid social events, restrict contact with friends and family, and find it difficult to complete their family or professional commitments, resulting in a heightened sensation of social isolation and a decline in quality of life [11]. With early detection and intervention for SIA, patients’ mental health can be effectively improved, and stable social interactions can be maintained.

In recent years, the role of systemic inflammatory immune index in the cancer symptom network has received increasing attention. Comprehensive indicators can more comprehensively reflect the inflammatory immune balance of the body. From a biological mechanisms perspective, studies have revealed that inflammatory factors can govern the connection between neuropathy and functional impairment, by influencing the sensitivity, neuroplasticity, and muscle metabolism of the central and peripheral nerve systems, consequently modulating pain perception and physical function [12]. The systemic inflammatory immune states, as exemplified by Neutrophil-to-Lymphocyte Ratio (NLR), may have a role in regulating peripheral nerve sensitivity and muscle metabolic processes via alterations in the balance of neutrophil-mediated oxidative stress and lymphocyte-mediated immune regulatory pathways [13]. Additionally, chemotherapy-induced neuroinflammation has been linked to the activation state of the NLRP3 inflammasome, which may affect neural plasticity and the transition of CIPN to limb dysfunction [14]. Therefore, we looked into any possible relationships between the immune-inflammatory index and CIPN and DASH in patients with breast cancer.

Although psychosocial issues among breast cancer patients have received significant attention, few studies have specifically examined the interactions between CIPN, DASH, and psychosocial distress in this population, and even fewer have included inflammatory markers in their analyses. This gap emphasizes the need for additional investigation. As a result, the purpose of this cross-sectional study is to investigate the factors associated with indicators of psychosocial problems in breast cancer patients, as well as to examine the relationship between CIPN and SIA, including the potential mediating role of DASH, while also testing whether NLR has a moderating effect on the relationship between CIPN and DASH. The studies aim to provide positive support measures to help breast cancer patients regain their mental health and social relationships.

### Theoretical framework and hypothesis formulation

According to the Biopsychosocial Model (BPSM), an individual’s health outcomes are controlled by the dynamic interplay and synergy of physiological, psychological, and social elements, rather than a single biological cause. While the therapeutic focus is on biological or medical issues in breast cancer patients, the psychological factor must always be considered [15]. Based on this model, the study proposes that chemotherapy-related physical symptoms in breast cancer patients may be linked to further physical and psychosocial impairments.

The World Health Organization developed the International Classification of Functioning, Disability, and Health (ICF), which is divided into two broad domains: functional and contextual. In terms of functioning, an individual’s health status includes physical structure and function, activity, and social participation [16]. Treating related symptoms of discomfort can easily lead patients to avoid physical activity, resulting in a deterioration in athletic ability and a drop in functional exercise [17].

These changes frequently make it more difficult to recover from physical disabilities, restrict patients’ social participation, and worsen social anxiety. Taking these perspectives into account, we developed a conceptual model that clarifies the links between CIPN, DASH, and social psychological disorders in breast cancer patients.

Furthermore, the ICF model incorporates both personal and environmental aspects into its contextual factors, with personal factors such as age, gender, and biological traits [18]. On this basis, this study included NLR as a moderating variable in the model.

Through this model, this study aims to provide preliminary theoretical foundations and empirical evidence to assist clinical healthcare professionals in developing targeted rehabilitation strategies for breast cancer patients, with a view to improving their long-term quality of life. Specifically, we propose the following hypotheses:

- H1: CIPN is positively correlated with DASH.
- H2: CIPN is positively correlated with SIA.
- H3: DASH is positively correlated with SIA.
- H4: DASH mediates the relationship between CIPN and SIA.
- H5: NLR moderates the relationship between CIPN and DASH.

## Methods

### Study design and participants

Convenient sampling was used to recruit 285 breast cancer patients at Wuhan University’s People’s Hospital between March 2025 and September 2025. This study is a cross-sectional study involving 16 independent variables. The sample size estimation is based on the traditional suggestion of using 5 to 10 times the independent variable, and considering a 20% invalid sample size [19]. The initial required sample size is calculated to be between 96 and 192 cases. And to ensure the stability of model testing, a sample size of at least 200 cases is required [20]. To ensure statistical rigour, this work used the G*Power 3.1 program to undertake a formal power analysis [21]. The heart of this study employs a PROCESS macro-mediation and moderated mediation model. The minimal sample size needed for this investigation was determined to be 207 cases using the typical moderate effect size of f² = 0.10 frequently used in clinical research, with 16 predictor variables, a significance level of α = 0.05, and a power of 1-β = 0.80. The necessary sample size is at least 249 cases, accounting for a 20% rate of erroneous data.

Inclusion criteria: Female patients with primary breast cancer confirmed by histopathology; aged ≥ 18years; without cognitive impairment or language communication difficulties; possessing primary school education or above, capable of independently completing questionnaires; patients who are informed about their condition and the study content, and voluntarily participate.

Exclusion criteria: Patients with other malignant tumors or combined major diseases of the heart, lungs, liver and kidneys; history of peripheral neuropathy (e.g., diabetic neuropathy, history of nerve injury, history of nerve surgery, etc.) or concomitant joint, muscular, or other disorders; history of limb surgery or functional impairment (numbness in the limbs caused by cervical and/or lumbar spine disorders, etc.); patients currently receiving treatment with other neurotoxic drugs; history of psychiatric disorders or severe trauma; refusal to participate or withdrawal during the study period.

### Ethical considerations

This study has been authorized by the Ethics Committee of Wuhan University People’s Hospital (approval number WDRY2025-K294). It was conducted in strict accordance with the ethical principles outlined in the Declaration of Helsinki, and informed consent was obtained from all subjects.

### Research tools

#### General information questionnaire

The general information questionnaire collected basic sociodemographic characteristics, including age, body mass index (BMI), education level, occupation, monthly income, medical insurance type, smoking history, alcohol history and disease-related clinical data such as chronic disease history (e.g., high blood pressure and diabetes, etc.), number of chemotherapy cycles, taxane-based drugs, platinum-based drugs, treatment modality, whether to have surgery, postoperative period, axillary lymph node dissection status, and NLR=neutrophil count/lymphocyte count.

#### Chemotherapy induced Peripheral Neuropathy Assessment Tool (CIPNAT)

The CIPNAT is an assessment tool for CIPN developed by American scholars Tofthagen et al. [22]. Wang Yue et al. [23]translated this scale into Chinese. The Chinese version of the scale produced content validity indices of 0.89-1 and Cronbach’s alpha coefficients of 0.891–0.941, indicating strong validity and reliability. This scale is divided into two sections: the first examines patients’ experiences with CIPN symptoms and consists of nine items. Each item measures symptom incidence, severity, distress caused, and frequency, for a total of 36 items with a possible score ranging from 0 to 279 points. The second portion comprises 14 elements that address the impact of CIPN on patients’ ability to perform daily tasks. A grading system with a range of 0 to 10 points is used, where 0 represents “no impact whatsoever,” and 10 represents “extremely affected.” The total score ranges from 0 to 140 points. A higher score indicates a larger impact of symptoms on day-to-day living. The Cronbach’s alpha coefficient for this scale is 0.968.

#### Quick Disabilities of the Arm, Shoulder and Hand Questionnaire (Quick DASH)

Although CIPN can affect both the upper and lower limbs, postoperative and chemotherapy-related functional impairments in breast cancer patients are most pronounced in the upper limbs (e.g., lymphoedema, restricted shoulder joint mobility, and impaired fine motor skills in the hands); thus, this study used the Quick DASH to assess upper limb-specific function. Beaton et al. [24]created the initial DASH scale, which had 31 elements, in 2001. In 2005, this was reduced to 11 items [25], it has been shown to have good reliability and validity, with a Cronbach’s alpha coefficient of 0.92 or higher and an intragroup correlation coefficient of 0.94 or higher. The abbreviated DASH scale was translated into Chinese in 2014 by Liao Chunli et al. [26].

The scale has two dimensions and a total of eleven items: Activities of Daily Life (6 items) and Upper Limb Symptoms (5 items). Items are assessed on a five-point scale: Daily Living Activities: No difficulty (1 point); Some difficulty (2 points); Moderate difficulty (3 points); Severe difficulty (4 points); Unable to accomplish (5 points). Upper Limb Symptoms: None (1 point), Mild (2 points), Moderate (3 points), Severe (4 points), and Extremely severe (5 points). The DASH scoring formula is: (patient score / number of items answered) − 1 × 25, resulting in a score range of 0 to 100. A higher score reflects more significant disability in the arm, shoulder, and hand region. The Cronbach’s alpha coefficient for this scale is 0.922.

#### Social Interaction Anxiety Scale (SIAS)

The 19-item SIAS scale, created by Mattick and Clarke in 1998, was used in this investigation [27]. It has proven to have good psychometric qualities in a variety of populations, with Cronbach’s alpha coefficients usually falling between 0.88 and 0.94. 

The Social Interaction Anxiety Scale (SIAS) has a 5-point Likert scale with 0 representing “Not at all compliant” and 4 representing “Fully compliant”, with items 8 and 10 reverse-scored. Higher scores indicate more severe SIA. These items are assessed in reverse. The Cronbach’s alpha coefficient for this scale is 0.945.

### Data collection process

To reduce potential bias, this study used a variety of techniques. To begin, all research team members were given uniform training to guarantee that the questionnaire was administered consistently and that information bias was minimised.

Before completing the questionnaire, participants provided prior agreement and were informed of the confidentiality principle. To ensure a consistent data collection process, the electronic questionnaire was administered face-to-face; researchers used standardised instructions to explain the completion procedure to patients and guided them to complete the questionnaire independently, without any leading prompts or assistance. The researchers gathered disease-related data from the clinical records system and double-checked the data to confirm its accuracy. After the questionnaires were reviewed and gathered on-site, 18 were eliminated due to systematic response patterns (e.g., choosing the same option for many consecutive questions, marking answers in a predetermined order or pattern, or completing the questionnaire in less than 300 s). A total of 303 questionnaires were distributed, with 285 valid surveys returned, for a response rate of 94.1%.

### Statistical analysis

SPSS version 27.0 was used for all data analysis. Categorical variables are shown as frequencies (N) and percentages. Continuous variables with a normal distribution are represented as mean ± standard deviation (M ± SD). Normality tests were run. For continuous data corresponding to dichotomous grouping variables, an independent samples t-test was used, and for continuous data relating to multi-category grouping variables, a one-way ANOVA was used. Pearson’s correlation coefficient was used to examine the associations between CIPN, DASH, SIA, and NLR. The mediating effect model of DASH in the link between CIPN and SIA was constructed using Model 4 of the SPSS PROCESS macro. The mediating and moderating influence model of the NLR was established using Model 7 of the same macro, which included the variables with *P* < 0.05 from the univariate analysis as confounding variables in Models 4 and 7 to account for them. With 5000 bootstrap resamples to estimate indirect effects and their 95% confidence intervals, the test level α = 0.05. All statistical analyses were considered significant at *P* < 0.05.

## Results

### Demographic and clinical disease-related characteristics of breast cancer patients

A total of 285 breast cancer patients were included, of which 61.8% were aged between 40 and 60 years old, with normal BMI (59.6%). Most patients used resident insurance, and most patients used taxane-based or platinum-based drugs. Table 1 summarizes the detailed features; the t, F, and *P* values in the table show the outcomes of group comparisons of SIA scores.

**Table 1:** Differences in SIA scores among breast cancer patients with different characteristics (*n* = 285)

Variable		*N*	%	t/F	*P*
Age (yr)					
	< 40	40	14.0	0.963	0.383
	40 ~ 60	176	61.8		
	> 60	69	24.2		
BMI (kg/m^2^)					
	<18.5	10	3.5	3.519	0.031
	18.5 ~ 24	170	59.6		
	≥ 24	105	36.8		
Education level					
	Primary school	74	26.0	7.268	< 0.001
	Junior high school	67	23.5		
	Senior high school/Technical secondary school	74	26.0		
	Bachelor’s degree/College diploma or above	70	24.6		
Occupation					
	Workers	46	16.1	2.283	0.047
	Farmers	57	20.0		
	Traders	12	4.2		
	Clerical staff	80	28.1		
	Freelancers	42	14.7		
	Unemployed	48	16.8		
Monthly income (￥)					
	<2500	101	35.4	6.082	< 0.001
	2500 ~ 5000	127	44.6		
	5001–7500	44	15.4		
	> 7500	13	4.6		
Medical insurance type					
	Resident insurance	163	57.2	3.583	0.029
	Employee insurance	114	40.0		
	Other	8	2.8		
Smoking history					
	Yes	12	4.2	3.566	< 0.001
	No	273	95.8		
Alcohol history					
	Yes	14	4.9	1.926	0.055
	No	271	95.1		
Chronic disease history					
	Yes	82	28.8	-0.541	0.589
	No	203	71.2		
Number of chemotherapy cycles					
	1 ~ 4	161	56.5	1.853	0.159
	5 ~ 8	118	41.4		
	>8	6	2.1		
Taxane-based drugs					
	Yes	213	74.7	0.281	0.779
	No	72	25.3		
Platinum-based drugs					
	Yes	149	52.3	1.363	0.174
	No	136	47.7		
Treatment modality					
	Neoadjuvant therapy	115	40.4	0.614	0.540
	Adjuvant therapy	170	59.6		
Whether to have surgery					
	Yes	199	69.8	-1.797	0.073
	No	86	30.2		
Postoperative period (month)					
	<3	96	48.2	1.218	0.304
	3 ~ 6	42	21.1		
	6 ~ 12	38	19.1		
	>12	23	11.6		
Axillary lymph node dissection status					
	Yes	129	64.8	0.846	0.399
	No	70	35.2		

*Yr *Year, *M *Mean, *SD *Standard Deviation, *BMI *Body Mass Index

### Descriptive and correlation analysis of the main variables in the mediation model

The following are the primary variables’ mean values and standard deviations in this study: 78.91 ± 51.73 for CIPN, 34.90 ± 19.33 for DASH, 19.91 ± 13.35 for SIA, and 2.71 ± 3.37 for NLR. Significant pairwise positive connections between CIPN, DASH, and SIA were found within the model using correlation analysis (*P* < 0.001). 

There was no significant link between NLR and CIPN (*r* = -0.030, *P* = 0.617) 、 DASH (*r* = 0.029, *P* = 0.627). The correlation coefficients were close to zero, indicating a very weak correlation. From a statistical standpoint, research has shown that this low correlation can significantly lower the danger of multicollinearity in moderation models, assuring model stability [28]. To illustrate the effect of a moderator, test the significance of the interaction term (CIPN × NLR). Even if NLR has no substantial correlation with CIPN or DASH, it can nevertheless act as a moderator. As demonstrated in Table 2.

**Table 2:** Variable descriptors and correlation analysis results

Variable	(M ± SD)	The correlation coefficient between variables			
		CIPN	DASH	SIA	NLR
CIPN	(78.91 ± 51.73)	1			
DASH	(34.90 ± 19.33)	0.336^**^	1		
SIA	(19.91 ± 13.35)	0.308^**^	0.225^**^	1	
NLR	(2.71 ± 3.37)	-0.030	0.029	-0.030	1

*M *Mean, *SD *Standard Deviation, *CIPN *Chemotherapy-induced Peripheral Neuropathy, *DASH *Disabilities of the Arm, Shoulder and Hand, *SIA *Social Interaction Anxiety, *NLR* Neutrophil-to-Lymphocyte Ratio

**P < 0.001

### The mediating role of DASH

To explore DASH’s potential mediating function in the link between CIPN and SIA, we conducted a mediation study using the PROCESS macro model 4. CIPN was used as the independent variable, SIA as the dependent variable, and DASH as the mediating variable. As shown in Table 3, a significant positive association was found between CIPN and SIA (β = 0.308, *P* < 0.001) before controlling for confounding variables. The direct association between CIPN and SIA decreased but remained significant (β = 0.262, *P* < 0.001) after DASH was included; additionally, DASH demonstrated a significant positive association (β = 0.137, *P* < 0.05). CIPN remained significantly positively associated with SIA (β = 0.279, *P* < 0.001) after controlling for confounding variables that were significant in univariate analysis, such as BMI, education level, occupation, monthly income, medical insurance type and smoking history. When DASH was added, the direct association between CIPN and SIA decreased but remained significant (β = 0.229, *P* < 0.001), while DASH also demonstrated a significant positive association with SIA (β = 0.143, *P* < 0.05), suggesting that the mediating effect of DASH remains robust. 

**Table 3:** Mediating effect analysis of DASH

Adjustment		Dependent variable	Independent variable			Fit index				Coefficient significance			
						R	R^2^			F	β	t	
Unadjusted		DASH		CIPN	0.336			0.113		36.088^**^	0.336		6.007^**^
		SIA		CIPN	0.308			0.095		29.721^**^	0.308		5.452^**^
		SIA		CIPN	0.334			0.112		17.740^**^	0.262		4.398^**^
				DASH							0.137		2.304^*^
Adjusted		DASH		CIPN	0.405			0.164		4.453^**^	0.352		6.215^**^
		SIA		CIPN	0.475			0.225		6.590^**^	0.279		5.112^**^
		SIA		CIPN	0.492			0.242		6.672^**^	0.229		3.954^**^
				DASH							0.143		2.481^*^

*CIPN *Chemotherapy-induced Peripheral Neuropathy, *DASH *Disabilities of the Arm, Shoulder and Hand, *SIA* Social Interaction Anxiety

*P < 0.05，**P < 0.001

To examine the significance of this mediating impact, we also used the Bootstrap approach (5000 resampled samples, 95% confidence interval), which revealed a significant mediating effect, as shown in Table 4. The total effect size of CIPN on SIA was 0.072 (95% CI: 0.044 to 0.100), and the direct effect size was 0.059 (95% CI: 0.030 to 0.088). The indirect effect was 0.013 (95% CI: 0.003 to 0.025), and the mediating effect accounted for 18.06% of the total effect. Because the unstandardised indirect effect is rather tiny, it is necessary to refer to the completely standardized indirect effect to determine the effect size. The results suggest that the standardised indirect impact is 0.051(95% CI: 0.011 to 0.095), which is in the small-to-moderate range and has certain clinical significance. These data indicate that DASH partially mediates the relationship between CIPN and SIA.

**Table 4:** Bootstrap test results for mediating effects

Mediation effect path	Beta‌	B	SE	95% CI		Ratio of effect(%)
				Lower	Upper	
Total effect	0.279	0.072	0.014	0.044	0.100	100
Direct effect	0.229	0.059	0.015	0.030	0.088	81.94
Indirect effect	0.051	0.013	0.006	0.003	0.025	18.06

*SE *Standard error, *CI *Confidence Interval, *Beta *Standardized Regression Coefficient, *B *Unstandardized Regression Coefficient

### The moderating mediating role of NLR

**Fig. 1:**
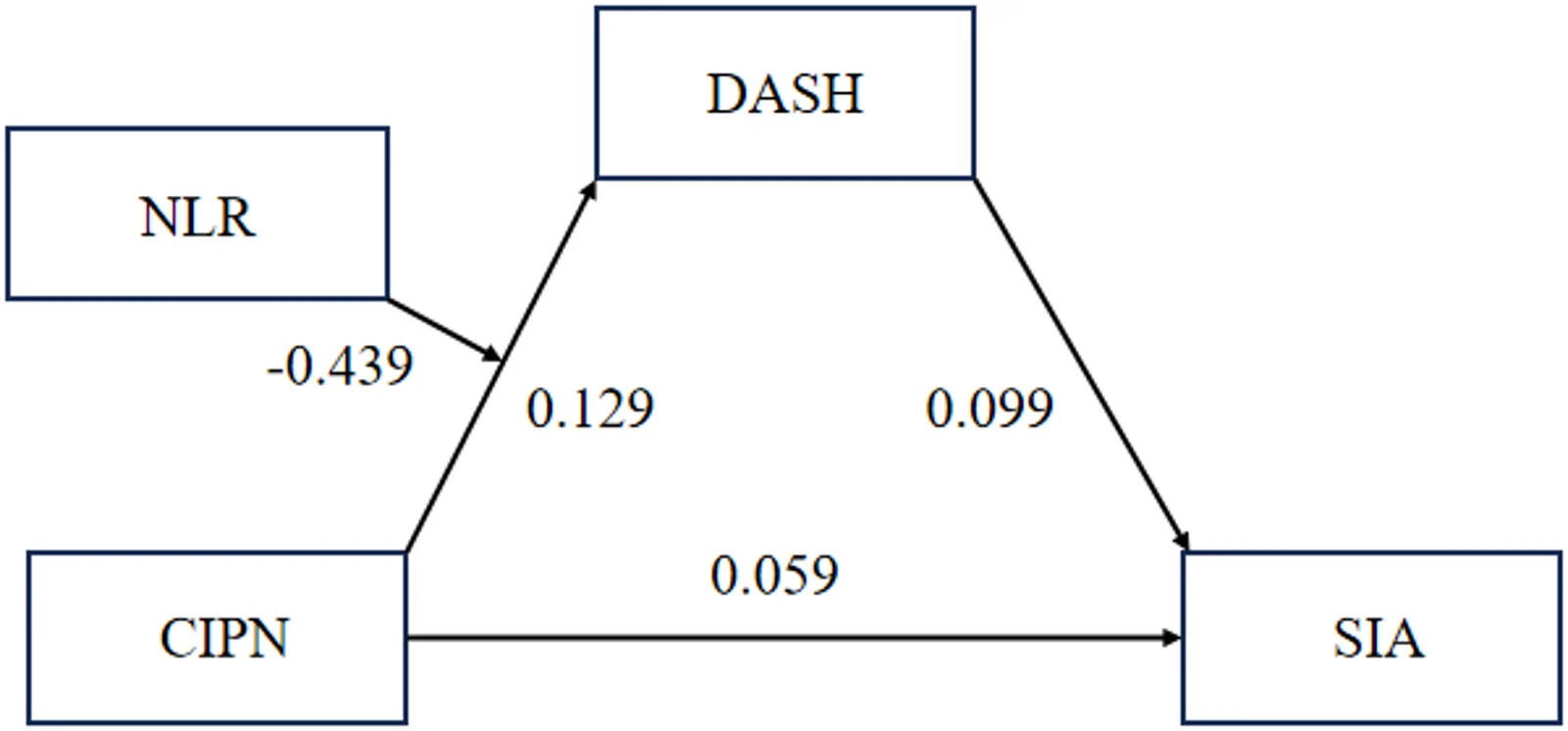
Influence pathway for the moderating role of NLR. (Note: CIPN=Chemotherapy-induced peripheral neuropathy，DASH=Disabilities of the arm, shoulder and hand，NLR=Neutrophil-to-lymphocyte ratio, SIA=Social Interaction Anxiety)

This study used the PROCESS macro model 7 to test whether NLR moderates the association between CIPN and DASH. The interaction term CIPN×NLR was significantly associated with DASH before adjusting for confounding variables (β = -0.023, t = -2.487, *P* = 0.013). After adjusting for confounding variables such as BMI, education level, occupation, monthly income, medical insurance type and smoking history, the interaction term CIPN×NLR remained significantly associated with DASH(β=-0.023, t=-2.501, *P* = 0.013). This model explained 18.6% of the overall variance in DASH; the results are presented in Table 5. These findings indicate that the NLR negatively moderates the association between CIPN and DASH, and that the strength of this association changes according to NLR levels. Figure 1 depicts the moderated mediation model framework established for this investigation, as well as the variables’ relationship paths.

**Table 5:** Path coefficient of the moderating effect

Adjustment	Dependent variable	Independent variable	Fit index		Coefficient significance			95% CI	
			R	R^2^	F	β	t	Lower	Upper
Unadjusted	DASH	CIPN×NLR	0.366	0.134	14.454^**^	-0.023	-2.487^*^	-0.042	-0.005
Adjusted	DASH	CIPN×NLR	0.432	0.186	4.415^**^	-0.023	-2.501^*^	-0.042	-0.005

*CIPN *Chemotherapy-induced Peripheral Neuropathy, *DASH *Disabilities of the Arm, Shoulder and Hand, *NLR *Neutrophil-to-Lymphocyte Ratio

^*^P < 0.05, ^**^P < 0.001

**Fig. 2:**
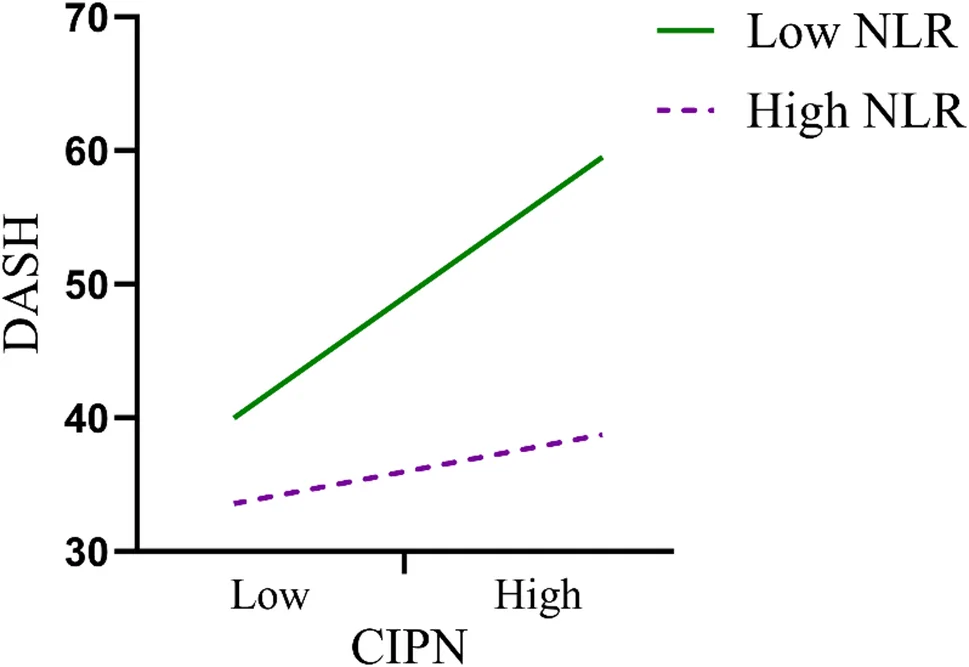
The moderating effect of NLR on the relationship between CIPN and DASH. (Note: CIPN=Chemotherapy-induced peripheral neuropathy, DASH=Disabilities of the arm, shoulder and hand, NLR=Neutrophil-to-lymphocyte ratio)

To further examine the moderating effect of the NLR, this study pre-processed it by centring it around the mean with the PROCESS macro. The NLR was split into three groups (low, medium, and high) based on M ± 1SD. A basic slope analysis was performed, as shown in Table 6. Figure 2 shows that the connection between CIPN and DASH was significant in the low NLR group (simple slope = 0.189, 95% CI: 0.128 to 0.250). The link was significant in the moderate NLR group (simple slope = 0.129, 95% CI: 0.088 to 0.171), but not in the high NLR group (simple slope = 0.050, 95% CI: -0.026 to 0.127). The slope trends in Fig. 2 clearly demonstrate this moderating effect: steeper slopes in the low NLR group imply a stronger influence of CIPN on DASH, while flatter slopes in the high NLR group correlate to a link that is no longer statistically significant.

**Table 6:** Analysis of NLR moderating effects

Path	NLR	Effect	Boot SE	95% CI	
				Lower	Upper
CIPN→DASH	-2.565 (M-1SD)	0.189	0.031	0.128	0.250
	0.000(M)	0.129	0.021	0.088	0.171
	3.372(M+1SD)	0.050	0.039	-0.026	0.127

*Boot SE *Bootstrap Standard Error‌, *CI *Confidence Interval, *M *Mean, *SD *Standard Deviation, *NLR *Neutrophil-to-Lymphocyte Ratio, *CIPN *Chemotherapy-induced Peripheral Neuropathy, *DASH* Disabilities of the Arm, Shoulder and Hand

## Discussion

This study constructed and validated a theoretical model, which showed that DASH played a partial mediating role between CIPN and SIA in Chinese female breast cancer patients. We also discovered that NLR negatively regulates the association between CIPN and DASH.

The average SIA score in our group suggests that breast cancer patients have a general level, which is lower than the findings published by Wang et al. [5]. This discrepancy may be due to differences in the timing of the surveys; that study focused mostly on post-operative breast cancer patients, and changes in body image have a substantial impact on SIA. It is worth mentioning that in the cross-sectional mediation model analysis, both CIPN and DASH were found to have a stable relationship with healthy social psychological levels.

There is a positive association between CIPN and SIA in breast cancer patients. This supports the findings of Verhoeff-Jahja et al. [29]. CIPN’s neurological impairment can cause discomfort and embarrassment for patients in situations such as shaking hands and social interactions, whereas symptoms such as limb weakness and difficulty walking directly affect patients’ physical mobility and willingness to participate in social activities, resulting in reduced social interaction. At the same time, neuropathic pain and sensory abnormalities can worsen negative psychological states like anxiety and depression [30], and some patients are unable to effectively manage the discomfort caused by their symptoms, making it difficult for them to actively maintain healthy social relationships [31]. These data imply that CIPN’s impact extends beyond the physical domain and may interact with psychosocial adaptation.

Importantly, the results of the cross-sectional mediation model clarified this link, suggesting that DASH functions as a mediator. This indirect effect accounts for 18.06% of the total association between CIPN and SIA, implying that CIPN’s impact on SIA may also be mediated through other pathways, such as the direct influence of sensory symptoms (numbness, tingling) on social situations [32], as well as the contribution of comorbid symptoms such as fatigue, pain, cognitive impairment, depression, and low social support [33–36]. Although the proportion of this mediating effect is not too high, its effect size cannot be ignored from a clinical standpoint. This DASH-mediated effect shows that physical rehabilitation training helps individuals with CIPN reduce functional impairment in their limbs. Existing research confirms that regular upper limb rehabilitation training can effectively enhance upper limb function and that functional improvements are significantly associated with improved social participation [37]. This pattern can be explained using the biopsychosocial model [16]; disease or treatment-related physical symptoms can influence an individual’s social role performance and interpersonal interactions via the primary pathway of functional limits, eventually leading to psychosocial issues. We have integrated key theoretical perspectives based on a cross-sectional mediation model: the direct effects model of CIPN, which emphasises CIPN’s direct role in the management of physical symptoms and psychosocial adaptation; and the functional limitation mediation model, which suggests that somatic sensory abnormalities may exacerbate psychosocial problems through physical disability. Future studies should not focus solely on the DASH pathway, but also consider CIPN’s sensory symptoms and other psychosocial aspects. Subsequent research should look into mediating variables beyond the DASH pathway to give a foundation for building a more comprehensive approach to controlling SIA.

In this study, we discovered that the relationship between CIPN and DASH was negatively regulated by NLR (β = −0.023, *P* < 0.05). It should be acknowledged that the interaction effect’s regression coefficient was tiny, indicating a weak moderating influence of NLR on the CIPN→DASH connection. A weak effect, however, does not always mean that it has no clinical significance. A single biomarker’s independent regulatory role is frequently limited within a complex network of symptoms; the relationship between CIPN and DASH is probably influenced by a combination of factors, and in this study, the NLR only explains a small percentage of the variation in DASH. From a clinical practice perspective, this weak effect suggests that NLR levels should not be relied upon alone to predict or intervene in limb dysfunction.

Interestingly, negative regulation suggests that an increase in systemic inflammation levels within a certain range may not simply exacerbate functional impairment, but may actually weaken the relationship between CIPN and DASH. This finding, which appears to contradict conventional wisdom, is thought to be closely related to the dual role of inflammation in both nerve damage and the body’s repair processes. According to conventional wisdom, neuropathic pain and functional impairment are mostly caused by inflammation [15, 38]. Other research, however, suggests that the inflammatory process can be both protective and harmful [39] and that tissue healing requires a mild inflammatory response, which may aid in injury recovery by triggering endogenous defenses. For example, upregulation of inflammatory cytokines (e.g., IL-9 and TGF-β) after nerve injury has been linked to pain relief and functional improvement [40]. A controlled inflammatory response is necessary for clearing necrotic tissue, initiating regeneration, and repairing muscle damage [41]. It should be clarified that the cross-sectional design of this study cannot determine causal relationships, and the effect size is minor. Further validation through large sample longitudinal studies is needed in the future. Although this effect is not yet sufficient to guide clinical decision-making adjustments at this stage, it provides a new perspective for further exploring the relationship between CIPN and limb dysfunction.

This study found that the NLR’s modulatory effect is only meaningful at low to moderate levels; at high levels, however, it no longer affects the relationship between CIPN and DASH. One possible explanation is that when inflammation is moderate, the immune system may be in a relatively plastic state, able to switch between pro-inflammatory and anti-inflammatory phenotypes in response to signals from the local microenvironment, thereby exerting regulatory effects [42]. We speculate that when the NLR is at a high level, intense systemic immune activation may disrupt immune homeostasis, and an overwhelming pro-inflammatory response may inhibit endogenous repair mechanisms [43]. At the same time, a continuous state of elevated inflammation can cause severe systemic symptoms such as weariness, malaise, and mental distress [44]. According to symptom competition theory, an individual’s attention and reporting resources are limited; more conspicuous and unpleasant systemic symptoms may lower their focus on and perception of CIPN-related local symptoms [45]. The above explanation is still speculative and requires additional clinical data to support it. Furthermore, statistically, the lack of a significant moderating impact in the high-level group could be attributed to the subgroup’s small sample size and higher variance. Future research could expand the sample size and undertake longitudinal studies to clarify causal relationships between variables, and test for a threshold influence in inflammation levels.

## Conclusion

This study suggests that there is a positive correlation between the occurrence of CIPN in breast cancer patients and their social interaction anxiety. In this study, mediating effect analysis was employed to confirm the direct and indirect relationship between CIPN and social interaction anxiety. DASH has a direct positive association with social interaction anxiety. On this basis, NLR negatively regulates the correlation strength between CIPN and DASH, but due to the extremely small effect size, we hope to further verify it in future research. In order to improve CIPN and lessen patients’ suffering, medical personnel should enhance the assessment and monitoring of SIA and DASH in breast cancer patients and create suitable and successful intervention programs. In order to deliver more individualized, comprehensive care, future focus should be on recognizing and resolving the relationship between patients’ subjective symptoms and psychosocial results.

## Limitations

There are still certain restrictions on this study. Convenience sample was used in this study, and each participant was selected from a single tertiary hospital. The use of convenience sampling brought potential selection bias, and the findings’ generalizability may be limited in terms of geographical scope and the promotion of the research results may have regional and population limitations, significantly reducing the external validity of the research results. Cross-sectional research cannot determine causal links between variables. Future longitudinal studies or intervention trials are needed to validate the model’s stability and directionality. Second, the study’s primary variables—CIPN and DASH, for example—were self-reported and did not include objective clinical physical tests. Although this approach records patients’ subjective experiences, subjective perception mistakes may compromise the accuracy of the findings. Future research could incorporate objective assessment metrics to further enhance the robustness of the results. At the same time, 74.7% of the study’s patients received taxane-based chemotherapy, whereas 52.3% received platinum-based drugs. The external validity of the study’s conclusions for non-Chinese populations and breast cancer patients receiving other treatment regimens is questionable, further restricting the findings’ generalizability. To validate the universality of these findings, future studies should increase the sample size and conduct multicenter, multi-population studies. 

## Data Availability

The datasets used and/or analysed during the current study are available from the corresponding author on reasonable request.
